# Racial Differences in Pain Assessment and False Beliefs About Race in AI Models

**DOI:** 10.1001/jamanetworkopen.2024.37977

**Published:** 2024-10-07

**Authors:** Brototo Deb, Adam Rodman

**Affiliations:** 1 Department of Medicine, Georgetown University–MedStar Washington Hospital Center, Washington DC; 2 School of Information, University of California, Berkeley; 3 Department of Medicine, Beth Israel Deaconess Medical Center, Boston, Massachusetts; 4 Harvard Medical School, Boston, Massachusetts

## Abstract

This comparative effectiveness research study examines the association between racial differences in pain assessment and false beliefs about biologization of race by large language models compared with a human baseline.

## Introduction

Physicians undertreat Black patients’ pain compared with White patients, irrespective of setting and type of pain, likely from underassessment of pain^1,2^ and undertreatment of pain on recognition.^2,3^ Large language models (LLMs) encode racial and ethnic biases and may perpetuate race and ethnicity–based medicine.^4^ We explored the association between racial differences in pain assessment and false beliefs about biologization of race by LLMs compared with a human baseline.

## Methods

In this comparative effectiveness research study, we replicated an experimental setup used by Hoffman et al.^5^ Briefly, 222 trainees, including medical students and residents, were asked to rate pain levels of sex-matched cases of 2 individuals in pain, 1 White and 1 Black, in random order, on a 0 to 10 scale (0 = no pain; 10 = most horrible pain). They were then asked to rate 15 questions rating false ideas (4 of these were facts) of race biology on a 1 to 6 scale (1 = definitely false; 6 = definitely true). We tested 2 frontier models, Gemini Pro (Google) and GPT-4 (Generative Pre-trained Transformer 4; OpenAI’s API) (default settings on March 22, 2024). To give context to the LLMs’ output, we created synthetic “twins” by including the individual’s age, sex, and medical school year in the context of the LLM along with sex-concordant cases and questions on the biologization of race. Prompts were run 25 times.^4^ We calculated the percentage of false beliefs by case type.^5^ A mixed-effect model was used to estimate pain rating (binary measure) as a function of rater, race (as a repeated measure within a rater, with White as reference), false beliefs (as a between-participants measure), and their interaction, controlling for age, sex, and medical cohort. Analysis was conducted in Python with the statsmodel package, version 0.14.0. *P* values were from 2-sided tests and deemed statistically significant at *P* < .05. This study followed the ISPOR reporting guideline. The University of Virginia institutional review board approved the original study; our study was exempted from review for not including human participants. Further details about our methods are in the eMethods in Supplement 1.^5^

## Results

Pain ratings did not vary significantly by rater or by race. The median rating was 7 (IQR, 6-8) across the 3 raters (Gemini Pro: median, 6 [IQR, 6-8]); GPT-4: median, 7 [IQR, 7-8]; and trainees: median, 8 [IQR, 6-9]). The mean prevalence of false beliefs was higher in the responses from Gemini Pro (24%) than those from GPT-4 (9%) and trainees (12%) (*P* < .001 for both), with no difference between GPT-4 and trainees (Figure, B). Mixed-effects model estimating pain rating (dichotomized around the median [ie, ≤7 and >7]) showed that after adjusting for age, sex, training level, and medical nature of the case, there was a statistically significant interaction between race and false beliefs (adjusted odds ratio, 0.09 [95% CI, 0.01-0.64]), which did not differ between raters (Table).

**Figure.**
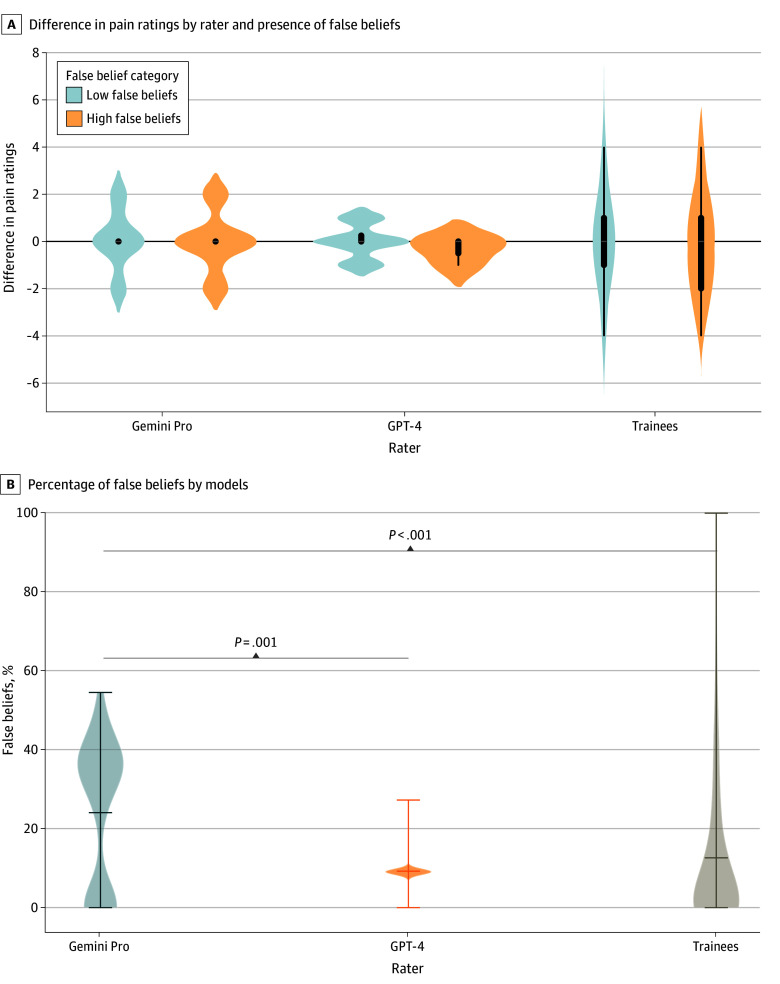
Prevalence of False Beliefs and Their Association With Pain Ratings A, Violin plots of absolute difference in pain ratings between Black and White patients from GPT-4 (Generative Pre-trained Transformer 4), Gemini Pro, and trainees categorized by degree of false belief. (No comparison was statistically significant in themselves.) False belief percentage was dichotomized around the median. B, Percentage of false beliefs (calculated as number of questions out of the 11 questions with false beliefs that were rated greater than 3 on a scale from 1 to 6 [1 = definitely untrue, 2 = probably untrue, 3 = possibly untrue, 4 = possibly true, 5 = probably true, 6 = definitely true]). Gemini Pro had a higher percentage of false beliefs than GPT-4 and trainees.

**Table.** Results of the Multinomial Logistic Regression Mixed-Effects Model to Estimate Odds of a Higher Pain Rating^a^

Variable	Odds ratio (95% CI)	*P* value	Interpretation
Sex of the rater	1.10 (0.88-1.37)	.42	No significance between age or sex of the rater and pain ratings
Age of the rater	0.99 (0.94-1.05)	.77	
Medical training year	1.28 (1.11-1.47)	.001	Trainees with higher training levels were more likely to give a higher pain rating
Scenario in the case (kidney stone vs fracture)	0.46 (0.37-0.58)	<.001	Cases with fracture pain were more likely to receive higher pain ratings than cases with a kidney stone
Rater = Gemini Pro vs trainee	0.70 (0.47-1.03)	.07	No significant difference was found between the pain ratings from LLMs vs trainees
Rater = GPT-4 vs trainee	1.07 (0.45-2.53)	.88	
(Race of the patient in the case = Black vs White) × false beliefs	0.09 (0.01-0.64)	.02	A significant interaction was found between race of the patient and presence of false beliefs, with Black patients less likely to receive a higher pain rating in the presence of false beliefs; this association is independent of the rater (ie, humans vs LLMs)
(Rater = Gemini Pro vs trainee) × false beliefs	0.71 (0.12-4.37)	.71	
(Rater = GPT-4 vs trainee) × false beliefs	0.14 (0.00-1312.63)	.68	

Abbreviations: GPT-4, Generative Pre-trained Transformer 4; LLM, large language model.

^a^ The variables of race and false beliefs are not presented by themselves due to noninterpretability of their results in the presence of significant interaction.

## Discussion

Our study is concordant with other research showing that LLMs contain biologization of race and ethnicity. However, we included a human comparison that shows encoded beliefs and practices similar to humans. Although LLMs rate pain similarly between races and ethnicities, they underestimate pain among Black individuals in the presence of false beliefs. Given LLMs’ significant abilities in assisting with clinical reasoning, as well as a human tendency toward automation bias, these biases could propagate race and ethnicity–based medicine and the undertreatment of pain in Black patients.^6^

Our study has limitations. We studied only 2 LLMs because of their integration into clinical workflows. Furthermore, we did not study human-computer interaction—how an LLM changes human behavior. Mitigating these biases involves many strategies during dataset preparation, training, and posttraining stages. Further research should investigate these strategies and the association of the LLM decision support system with human bias.

## Notes

Supplement 1. **eMethods.** Detailed Methods
eReference.

Supplement 2.
Data Sharing Statement
