# Supplementary material for: Racial Differences in Pain Assessment and False Beliefs About Race in AI Models
Source: JAMA Netw Open. 2024 Oct 7;7(10):e2437977. doi: 10.1001/jamanetworkopen.2024.37977 (PMC11459244; doi:10.1001/jamanetworkopen.2024.37977)
Supplement: Supplement 2. — Data Sharing Statement [file jamanetwopen-e2437977-s002.pdf]

Data Sharing Statement

Deb. Racial Differences in Pain Assessment and False Beliefs About Race in AI Models. *JAMA Netw Open*. Published October 07, 2024. doi:10.1001/jamanetworkopen.2024.37977

Data

Data available: The data for the study is publicly available from https://osf.io/crxwa/
